# Successful treatment of a unique case of solitary primary iliopsoas abscess caused by *Streptococcus dysgalactiae* subspecies *equisimilis*: A case report

**DOI:** 10.1097/MD.0000000000037602

**Published:** 2024-03-29

**Authors:** Yuichiro Fuchita, Hirokazu Toyoshima, Chiaki Ishiguro, Hiroyuki Tanaka

**Affiliations:** a Department of Pharmacy, Japanese Red Cross Ise Hospital, Ise, Japan; b Department of Infectious Diseases, Japanese Red Cross Ise Hospital, Ise, Japan; c Infection Prevention and Control Office, Japanese Red Cross Ise Hospital, Ise, Japan; d Department of Medical Technology, Japanese Red Cross Ise Hospital, Ise, Japan.

**Keywords:** case report, creatinine clearance, drainage, solitary primary iliopsoas abscess, *Streptococcus dysgalactiae* subspecies *equisimilis*

## Abstract

**Rationale:**

Iliopsoas abscess, mainly caused by *Staphylococcus aureus*, occurs via the bloodstream or spread from adjacent infected organs. Although a few cases regarding primary iliopsoas abscess caused by *Streptococcus dysgalactiae* subspecies *equisimilis* (SDSE) with accompanying disseminated foci have been reported to date, there has been no case report on solitary primary iliopsoas abscess caused by SDSE.

**Patient concerns:**

An 85-year-old Japanese woman presented with worsening right hip pain and fever after an exercise. Hip computed tomography revealed a right iliopsoas abscess (iliac fossa abscess), and intravenous cefazolin was started as a treatment based on the creatinine clearance level on admission.

**Diagnoses:**

Blood cultures were positive for β-hemolytic Lancefield group G gram-positive cocci arranged in long chains, which were identified as SDSE by matrix-assisted laser desorption/ionization. No other disseminated foci were found upon performing whole computed tomography and transthoracic echocardiography. The patient was diagnosed with an SDSE solitary iliopsoas abscess.

**Interventions:**

The antimicrobial was appropriately switched to intravenous ampicillin on day 2, with the dosage adjusted to 2 g every 6 hours based on the preadmission creatinine clearance, followed by oral amoxicillin (1500 mg, daily).

**Outcomes:**

The abscess disappeared without drainage on day 39, and the patient remained disease-free without recurrence or sequelae during a 6-month follow-up period.

**Lessons:**

SDSE can cause a solitary primary iliopsoas abscess, which can be successfully treated with an appropriate dose of antimicrobials without draining the abscess.

## 1. Introduction

Iliopsoas abscess is a collection of pus in the iliopsoas compartment and is classified as primary iliopsoas abscess, mainly caused by hematogenous seeding from a distant site, and secondary iliopsoas abscess, which occurs due to underlying diseases, including adjacent vertebral osteomyelitis.^[1]^ *Staphylococcus aureus* is the most common pathogen isolated from over 88% of patients with primary iliopsoas abscess; meanwhile, *Streptococcus dysgalactiae* subspecies *equisimilis* (SDSE) has rarely been isolated from patients with primary iliopsoas abscess.^[1]^ Moreover, SDSE, belonging to Lancefield groups C and G, can cause skin and soft-tissue infections, intraabdominal and epidural abscesses, and infective endocarditis in immunocompromised patients, such as those with diabetes mellitus.^[2]^ Previous cases of iliopsoas abscess caused by SDSE have been accompanied by other disseminated foci.^[3,4]^

To the best of our knowledge, there has been no case report of solitary primary iliopsoas abscess caused by SDSE; therefore, the pathogenesis and appropriate therapeutic strategy for such patients remain uncertain. Here, we describe an immunocompetent patient with a solitary iliopsoas abscess caused by SDSE infection and the treatment regimen administered to the patient.

## 2. Case

An 85-year-old Japanese woman was admitted to our hospital because of aggravating right hip pain and fever after performing an exercise of lifting both legs while lying on the back 2 days ago. Her height and weight at the first visit were 153 cm and 66.4 kg, respectively (body mass index: 28.4 kg/m^2^). She had undergone bilateral total hip arthroplasty for hip osteoarthritis 20 years ago and had a history of mitral regurgitation, hypertension, atrial fibrillation, and chronic kidney disease with 22.9 mL/min of creatinine clearance (CrCL). She took apixaban (5 mg, daily) as her regular medication for atrial fibrillation.

**Figure 1.**
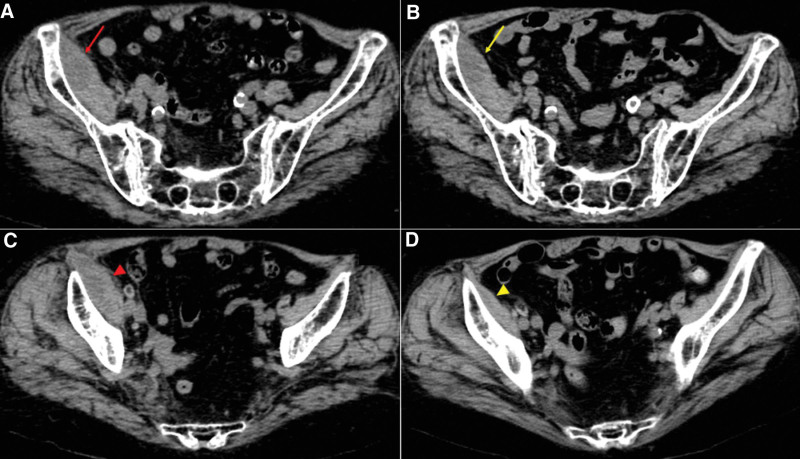
(A and B) Computed tomography (CT) on admission revealed a solitary right iliac fossa abscess sized 1.9 cm × 6.0 cm × 5.2 cm (red arrow and arrowhead) (A, sacroiliac level; B, lower sacral level). (C and D) A follow-up CT on day 16 showed a shrinking abscess (yellow arrow and arrowhead) (A, sacroiliac level; B, lower sacral level).

On admission, she was alert and her vital signs were as follows: body temperature, 38.0 °C; blood pressure, 105/45 mm Hg; heart rate, 76 beats/min; respiratory rate, 16 breaths/min; and oxygen saturation, 90% on ambient air. Physical examination showed that passive extension of the right hip joint caused pain and she had bilateral leg edema without an apparent wound. Laboratory findings showed an elevation of White blood cell (WBC) count with a left shift of neutrophils, a high C-reactive protein (CRP) level, a high erythrocyte sedimentation rate (ESR), normocytic anemia, and renal dysfunction (Table 1). Hip computed tomography (CT) revealed a right iliopsoas abscess (iliac fossa abscess) sized 1.9 cm × 6.0 cm × 5.2 cm without disseminated foci in other sites (Fig. 1A and B). Transthoracic echocardiography showed no evidence of infective endocarditis.

**Table 1:** Laboratory data.

Hematology				Biochemistry			
WBC	16,300	/μL	(RR, 3800–9000/μL)	TP	5.4	g/dL	(RR, 6.7–8.3 g/dL)
Neut	91.9	%	(RR, 40.0%–75.0%)	Alb	2.7	g/dL	(RR, 3.9–4.9 g/dL)
Hb	9.0	g/dL	(RR, 11.0–15.0 g/dL)	AST	14	U/L	(RR, 8–38 U/L)
MCV	91.0	fL	(RR, 79.0–99.0 fL)	ALT	7	U/L	(RR, 4–44 U/L)
Plt	12.9	×10^4^/µL	(RR, 15.0–40.0 × 10^4^/µL)	γ-GTP	22	U/L	(RR, 13–51 U/L)
Coagulation tests				T-bil	0.5	mg/dL	(RR, 0.2–1.2 mg/dL)
PT-INR	1.24		(RR, 0.88–1.12)	LDH	151	U/L	(RR, 124–222 U/L)
APTT	33	s	(RR, 24–39 s)	BUN	75	mg/dL	(RR, 8–20 mg/dL)
ESR	110	mm/h	(RR, 2–10 mm/h)	Cr	3.77	mg/dL	(RR, 0.40–0.80 mg/dL)
Urinalysis				CK	130	U/L	(RR, 43–165 U/L)
Protein	±			CRP	330.0	mg/L	(RR, 0.0–1.0 mg/L)
OB	±			BG	101	IU/mL	(RR, 56–109 mg/L)
NAG	37.9	U/gCr	(RR, 1.0–6.3 U/gCr)	RF	1.0	IU/mL	(RR, ≤15.0 IU/mL)
BMG	1271.0	μg/L	(RR, 12–346 μg/L)	CH50	64.9	U/mL	(RR, 31.6–57.6 U/mL)
Urine sediment				BMG	7.80	mg/L	(RR, ≤2.00 mg/L)
Epithelial cast	<1	HPF					
Hyaline cast	1–4	HPF					

Alb = albumin, ALT = alanine transferase, APTT = activated partial thromboplastin time, AST = aspartate aminotransferase, BG = blood glucose, BMG = β2-microglobulin, BUN = blood urea nitrogen, CH50 = total complement activity, CK = creatine kinase, Cr = creatinine, CRP = C-reactive protein, ESR = erythrocyte sedimentation rate, γ-GTP = γ-glutamyltransferase, Hb = hemoglobin, HPF = high power field, LDH = lactate dehydrogenase, MCV = mean corpuscular volume, NAG = N-acetyl-β-D-glucosaminidase, Neut = neutrophils, OB = occult blood, Plt = platelet, PT-INR = prothrombin time-international normalized ratio, RF = rheumatoid factor, RR = reference range, T-bil = total bilirubin, TP = total protein, WBC = white blood cells.

**Figure 2.**
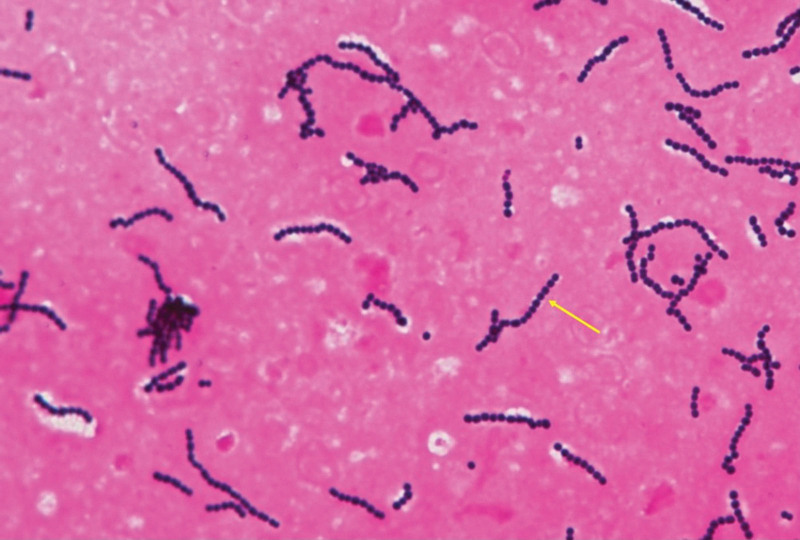
Gram staining (×1000) of the blood cultures revealed gram-positive cocci arranged in long chains (yellow arrow).

The patient was administered intravenous cefazolin (0.5 g every 12 hours), as initially methicillin-susceptible *S aureus* was suspected as the causative agent of the iliopsoas abscess. Two blood cultures were obtained, and after incubating the cultures for 11 hours, gram-positive cocci arranged in long chains were detected using the BacT/Alert system (bioMérieux, Marcy I’Etoile, France) (Fig. 2). The isolates were grown on 5% sheep blood agar (Nihon Becton Dickinson, Tokyo, Japan) and showed β-hemolysis with Lancefield group G antiserum (Kanto Chemical Co., Inc., Tokyo, Japan). The pathogen was identified as *S dysgalactiae* with a high score value (>2.0) using matrix-assisted laser desorption/ionization (MALDI Biotyper; Bruker Daltonik GmbH, Bremen, Germany).

The patient was diagnosed with a solitary iliopsoas abscess caused by SDSE. The isolates were found to be susceptible to penicillin using the VITEK 2 system (bioMérieux) (Table 2). The antimicrobial treatment was appropriately switched to intravenous ampicillin (2 g every 8 hours) on day 2 when the acute kidney injury (AKI) recovered to 22.8 mL/min, as measured using the Cockcroft–Gault formula. The patient became apyrexial on day 3, with gradual relief of right leg pain. Two sets of follow-up blood cultures on day 4 were negative for SDSE. Drainage of the abscess was not performed, considering the risk of hematoma due to apixaban. A follow-up CT on day 16 showed a shrinking abscess (Fig. 1C and D). The abscess disappeared without drainage on day 39 after intravenous administration of ampicillin for 10 days, followed by oral amoxicillin (1500 mg, daily) for 4 weeks. The patient remained disease-free without recurrence or sequelae during a 6-month follow-up period.

**Table 2:** Antimicrobial susceptibility test results.

*Streptococcus dysgalactiae* subspecies *equisimilis*		
Antimicrobials	MIC (µg/mL)	*Susceptibility
Penicillin	≤0.06	S
Ampicillin	≤0.25	S
Cefotaxime	≤0.12	S
Erythromycin	≥8	R
Clindamycin	≥1	R
Vancomycin	0.5	S
Linezolid	≤2	S

MIC = minimum inhibitory concentration, R = resistant, S = susceptible.

* Interpreted according to the Clinical and Laboratory Standards Institute criteria (Documents M100-Ed32).

## 3. Discussion

This report illustrates 2 clinical themes: the potential of SDSE to cause solitary iliopsoas abscess and the appropriate antimicrobial strategy to treat such patients with or without draining the iliopsoas abscess.

SDSE, typically β-hemolytic, may express Lancefield group C or G antigens.^[2]^ The mortality rate of SDSE bacteremia has been reported to be 15% to 18%,^[5]^ whereas that of *S aureus* bacteremia is 34%.^[6]^ Because the iliopsoas muscle has enriched blood supply, a bloodstream infection can easily affect this muscle.^[1]^ Primary iliopsoas abscess has been reported to be caused by *S aureus* mainly (88%); in contrast, *Streptococcus* species have rarely been reported to cause primary iliopsoas (4.8%).^[7]^ A PubMed search using the Medical Subject Headings “Psoas abscess” and “*Streptococcus dysgalactiae*” with the Boolean operator “AND” found only 2 articles in English each describing a case of iliopsoas abscess caused by *S dysgalactiae* (Table 3).^[3,4]^ We excluded case reports regarding infections caused by group C or G streptococci, as they may include not only *S dysgalactiae* but also other streptococci, such as *S. anginosus*. In both the cases of iliopsoas abscess we found,^[3,4]^ blood cultures were positive for SDSE, and there were other sites of SDSE infection in addition to SDSE iliopsoas abscess. A retrospective, multicenter surveillance study in Japan reported 6 cases of iliopsoas abscess with SDSE bacteremia.^[8]^ All cases included other sites of infections: 2 cases with vertebral osteomyelitis and 1 case with each of the following co-infections: cellulitis, septic arthritis, vertebral osteomyelitis pyogenic lymphadenitis, and vertebral osteomyelitis and urinary tract infection. To the best of our knowledge, the present study is the first case report on solitary iliopsoas abscess caused by SDSE. Thus, SDSE should be considered a causative microorganism even in cases of solitary iliopsoas abscess.

**Table 3:** Iliopsoas abscess caused by *Streptococcus dysgalactiae* subspecies *equisimilis*.

Case	Age (yr)	Sex	Predisposing factors	Symptoms	Sites of infections	Blood cultures	Inflammatory markers	Intervention	Antimicrobial duration (wk)	Outcome
Case 1^[3]^	63	M	Hyperuricemia Intramuscular injection (right buttock)	Fever Right hand inflammation Lumbar pain	Spondylodiscitis (T4–S1) Gluteal and right psoas abscess Multifocal septic arthritis	Positive	WBC 34,100/µL CRP 365 mg/L	Drainage	10	A
Case 2^[4]^	92	M	Hypertension Paroxysmal atrial fibrillation Polymyalgia rheumatica	Fever	Spondylodiscitis (L4–L5) Left psoas abscess Cellulitis of limbs Device-related endocarditis	Positive	WBC 20,000/µL CRP 302 mg/L	Aspiration	Lifelong	A

A = alive, CRP = C-reactive protein, F = female, M = male, WBC = white blood cell.

Iliopsoas abscesses are classified as primary, caused by bacteremia, or secondary, caused by bacterial spread from an infected organ.^[1]^ The patient was diagnosed with a primary iliopsoas abscess because the 2 sets of blood cultures of the patient were positive for SDSE and the CT scan did not find any infected organ. The most common causative microorganism for a primary iliopsoas abscess is *S aureus*, whereas pathogens responsible for a secondary iliopsoas abscess are more commonly from the gastrointestinal and genitourinary tracts, such as *Escherichia coli, Klebsiella* species, and *Streptococcus* species.^[9]^ The mortality rate of patients with gas-forming iliopsoas abscesses, in which gram-negative rods are predominant, is higher than that of patients with non-gas-forming iliopsoas abscesses, in which gram-positive cocci are predominant.^[10]^ Additionally, advanced age, WBC count, platelet count, blood urea nitrogen, creatinine, potassium, and secondary iliopsoas abscesses have been reported to be associated with the mortality rate of patients with iliopsoas abscesses.^[10]^ Tabrizian et al^[11]^ reported that patients with bacteremia and small abscess (<3.5 cm) responded well to antimicrobial therapy alone. In gas-forming or non-gas-forming cases without contraindication for surgical intervention, surgical intervention, including percutaneous drainage or operation, remains the first choice for treating iliopsoas abscesses.^[10]^ In the cases where *S dysgalactiae* was the causative agents of iliopsoas abscess,^[3,4]^ the patients underwent drainage or aspiration in addition to antimicrobial administration (Table 3). However, considering the contraindication of CT-guided drainage and the result of follow-up blood cultures, our patient was treated with intensive intravenous ampicillin for 10 days, followed by 4 weeks of oral amoxicillin administration. As a result, the abscess disappeared without drainage on day 39. This suggests that large solitary iliopsoas abscesses caused by SDSE bacteremia can be treated with antimicrobial therapy alone without needing to drain the abscess. The high blood supply to the iliopsoas muscle may have contributed to our case’s excellent response to antimicrobial therapy.

Generally, inflammation markers in patients with myalgia and fever should be evaluated,^[12]^ as inflammation markers such as WBC, CRP, and ESR are often elevated in patients with iliopsoas abscesses.^[1]^ The duration of intravenous antimicrobial administration is generally 2 weeks, followed by oral antimicrobials for 4 to 6 weeks, depending on inflammatory marker levels, clinical improvement, and follow-up CT results.^[13]^ The patient was treated with intravenous ampicillin for 10 days and oral amoxicillin for 4 weeks, according to WBC count, CRP, ESR, clinical improvement, and follow-up CT findings, without draining the abscess.

The antimicrobial dosage adjustment based on creatinine prior to admission may contribute to the success of the treatment. One-fifth of patients with acute infections develop AKI on admission; however, more than 50% of the patients with AKI have a resolution of renal injury by 48 hours.^[14]^ Therefore, during that time, the dose of beta-lactam drugs with a comprehensive safety zone should not be reduced.^[14]^ Antimicrobials with a wide safety margin, such as β-lactam and β-lactam/β-lactamase inhibitor combinations, allow dose adjustments to be deferred until 48 hours after the initiation when the patient’s renal function is better characterized.^[14]^ In addition, appropriate antimicrobial treatment for community-onset bacteremia within 48 hours is negatively correlated with the mortality rate.^[15]^ Our patient was treated with cefazolin as an empirical therapy with the dosage designed based on the CrCL on admission. However, the antimicrobial was switched to ampicillin the following day, with the dosage adjusted based on the preadmission CrCL. In our case, CrCL on day 2 improved from 11.4 to 22.8 mL/min; therefore, the patient was diagnosed with reversible AKI and did not require the readjustment of the antimicrobial dosage. Clinicians should pay attention to the information on preadmission CrCL for appropriate antimicrobial administration.

A limitation includes that SDSE was not directly proven as the causative organism of the iliopsoas abscess because of the high risk of hematoma.

In conclusion, SDSE can cause a solitary iliopsoas abscess. Clinicians should remain aware that the abscess caused by SDSE bacteremia can be treated with an appropriate dose of antimicrobials and may not require drainage.

## Acknowledgments

We would like to thank Editage (http://www.editage.com) for editing and reviewing this manuscript for English language.

## Author contributions

**Conceptualization:** Yuichiro Fuchita, Hirokazu Toyoshima, Chiaki Ishiguro, Hiroyuki Tanaka.

**Data curation:** Hirokazu Toyoshima, Chiaki Ishiguro.

**Methodology:** Yuichiro Fuchita, Hirokazu Toyoshima, Chiaki Ishiguro, Hiroyuki Tanaka.

**Supervision:** Hirokazu Toyoshima.

**Visualization:** Yuichiro Fuchita, Hirokazu Toyoshima.

**Writing – original draft:** Yuichiro Fuchita, Hirokazu Toyoshima.

**Writing – review & editing:** Yuichiro Fuchita, Hirokazu Toyoshima.
